# Optimising Ankle Foot Orthoses for children with Cerebral Palsy walking with excessive knee flexion to improve their mobility and participation; protocol of the AFO-CP study

**DOI:** 10.1186/1471-2431-13-17

**Published:** 2013-02-01

**Authors:** Yvette L Kerkum, Jaap Harlaar, Annemieke I Buizer, Josien C van den Noort, Jules G Becher, Merel-Anne Brehm

**Affiliations:** 1 Department of Rehabilitation Medicine, VU University Medical Center Amsterdam, De Boelelaan 1117, 1081 HV, Amsterdam, The Netherlands; 2 Research Institute MOVE, VU University Medical Center, Amsterdam, The Netherlands

**Keywords:** Cerebral Palsy, Pediatrics, Orthotic devices, Ankle foot orthoses, Intervention studies, Treatment effectiveness, Mobility, Participation, Gait

## Abstract

**Background:**

Ankle-Foot-Orthoses with a ventral shell, also known as Floor Reaction Orthoses (FROs), are often used to reduce gait-related problems in children with spastic cerebral palsy (SCP), walking with excessive knee flexion. However, current evidence for the effectiveness (e.g. in terms of walking energy cost) of FROs is both limited and inconclusive. Much of this ambiguity may be due to a mismatch between the FRO ankle stiffness and the patient’s gait deviations.

The primary aim of this study is to evaluate the effect of FROs optimised for ankle stiffness on the walking energy cost in children with SCP, compared to walking with shoes alone. In addition, effects on various secondary outcome measures will be evaluated in order to identify possible working mechanisms and potential predictors of FRO treatment success.

**Method/Design:**

A pre-post experimental study design will include 32 children with SCP, walking with excessive knee flexion in midstance, recruited from our university hospital and affiliated rehabilitation centres. All participants will receive a newly designed FRO, allowing ankle stiffness to be varied into three configurations by means of a hinge. Gait biomechanics will be assessed for each FRO configuration. The FRO that results in the greatest reduction in knee flexion during the single stance phase will be selected as the subject’s optimal FRO. Subsequently, the effects of wearing this optimal FRO will be evaluated after 12–20 weeks. The primary study parameter will be walking energy cost, with the most important secondary outcomes being intensity of participation, daily activity, walking speed and gait biomechanics.

**Discussion:**

The AFO-CP trial will be the first experimental study to evaluate the effect of individually optimised FROs on mobility and participation. The evaluation will include outcome measures at all levels of the International Classification of Functioning, Disability and Health, providing a unique set of data with which to assess relationships between outcome measures. This will give insights into working mechanisms of FROs and will help to identify predictors of treatment success, both of which will contribute to improving FRO treatment in SCP in term.

**Trial registration:**

This study is registered in the Dutch Trial Register as NTR3418.

## Background

With an incidence of 2–3 per 1000 living births, Cerebral Palsy (CP) is the most frequent cause of motor disorders in childhood in Western countries [1]. Spastic motor disorders are most common in children with CP, with symptoms of spasticity, muscle weakness and decreased selective motor control [2], often causing limitations in mobility [3], which may lead to a restricted participation in everyday life [4].

Although more than half of all children with bilateral spastic CP (SCP) walk independently with or without an assistive device [5], most experience mobility-related problems, such as reduced gait speed and/or an increased walking energy cost [6-12]. These problems are often caused by gait deviations [13-16], which can be corrected by prescribing ankle-foot orthoses (AFOs). An AFO imposes a mechanical constraint on the ankle, either to compensate for loss of function [17-19] or to counteract an excess of function [20,21]. An AFO therefore acts by applying control to the ankle and foot and, dependent on its design, it can indirectly stabilise the knee and hip joints [22]. As such, AFOs aim to improve, i.e. normalise joint kinetics, joint kinematics and spatio-temporal parameters [17,23-26]. Improvements in joint kinetics and kinematics have been shown to be closely coupled to an improved walking energy cost, which leads to benefits in walking ability; an effect also noted in the context of orthotic interventions [23,25-27]. This applies especially to children who walk with excessive knee flexion in midstance, since this walking pattern is particularly energy consuming [9,10] and these children are liable to show deterioration in walking ability in (pre-) puberty [28,29].

A variety of AFO types are available, depending on the specific gait deviations of the child. For children who walk with excessive knee flexion, orthoses with a ventral shell, also known as Floor Reaction Orthoses (FROs), are commonly prescribed [20]. Although FROs are widely used in SCP, evidence supporting their effectiveness is so far lacking. The decision-making process leading to FRO prescription is still based on expert opinion and experience (i.e. a trial-and-error approach), resulting in differences in treatment paradigms with respect to both the indication and the mechanical construction of FROs [30,31]. This is reflected in current literature, as studies have shown that wearing an FRO can be effective in decreasing walking energy cost, but may also have no effect [32] or even be adverse in some children in terms of walking energy cost or gait speed [26,32].

This variation in FRO effectiveness might be partly explained by the match of the mechanical properties of the orthosis to a patient’s specific gait deviations. Research in adults with neurological disorders has shown that walking energy cost with a typical spring-like AFO could be optimised by choosing the correct AFO ankle stiffness [33], suggesting that there may be an optimal match between a patient’s characteristics and the mechanical properties of an AFO. A similar principal might also apply to FROs.

A conventional FRO is a rigid type of AFO, and includes a ventral shell and a rigid footplate. The biomechanical mechanism of an FRO is to create a knee-extensor moment during midstance and terminal stance, by shifting of the ground reaction force forward [21]. Although an FRO might be effective in this respect, ankle push-off power is obstructed by an impeded plantar flexion in terminal stance and preswing. To enhance push-off power, a more spring-like FRO could potentially be beneficial, since it could store energy at the beginning of the stance phase that is released and returned in preswing. Achieving a sufficiently high stiffness to counteract knee flexion while including the potential benefit of spring-like properties in terms of walking energy cost may result in an optimal FRO stiffness based on the least compromise between these two goals.

Designing and evaluating the efficacy of such an optimal FRO requires an evaluation of the effects of different degrees of FRO ankle stiffness on various aspects of gait, i.e. function, mobility and participation. This implies a need for a set of outcome measures that covers all domains of the International Classification of Functioning, Disability and Health (ICF) [34]. Evaluating the effects of an intervention on more than one of the ICF domains will provide insights into mutual relations, thereby aiming to identify possible working mechanisms [35], which will contribute to improved FRO treatment.

FRO treatment could be further improved by identifying those children who could benefit from FROs [30]. Rogozinski et al. [21] explored clinical examination parameters that might explain the efficacy of FROs in CP children walking with excessive knee flexion. They found a strong, negative correlation between knee and hip flexion contractures and peak knee extension, achieved during walking with an FRO. Other studies have shown that child characteristics and environmental factors predict the response to rehabilitation interventions, such as Botulinum toxin A injections [36-38] and surgery [39-41]. Specific patient characteristics might also be relevant predictive factors for FRO efficacy.

In summary, evidence supporting the efficacy of FROs in children with SCP walking with excessive knee flexion remains inconclusive. Understanding of both the underlying working mechanisms and the factors predictive of treatment success is still lacking. Therefore, this project has two main goals:

1. To study the effect of an FRO optimised for ankle stiffness on walking energy cost in children with SCP walking with excessive knee flexion, compared to walking with shoes alone.

2. To identify the possible working mechanisms of an FRO, and the predictors for success of FRO treatment in children with SCP, walking with excessive knee flexion.

## Methods

### Design

**Figure 1:**
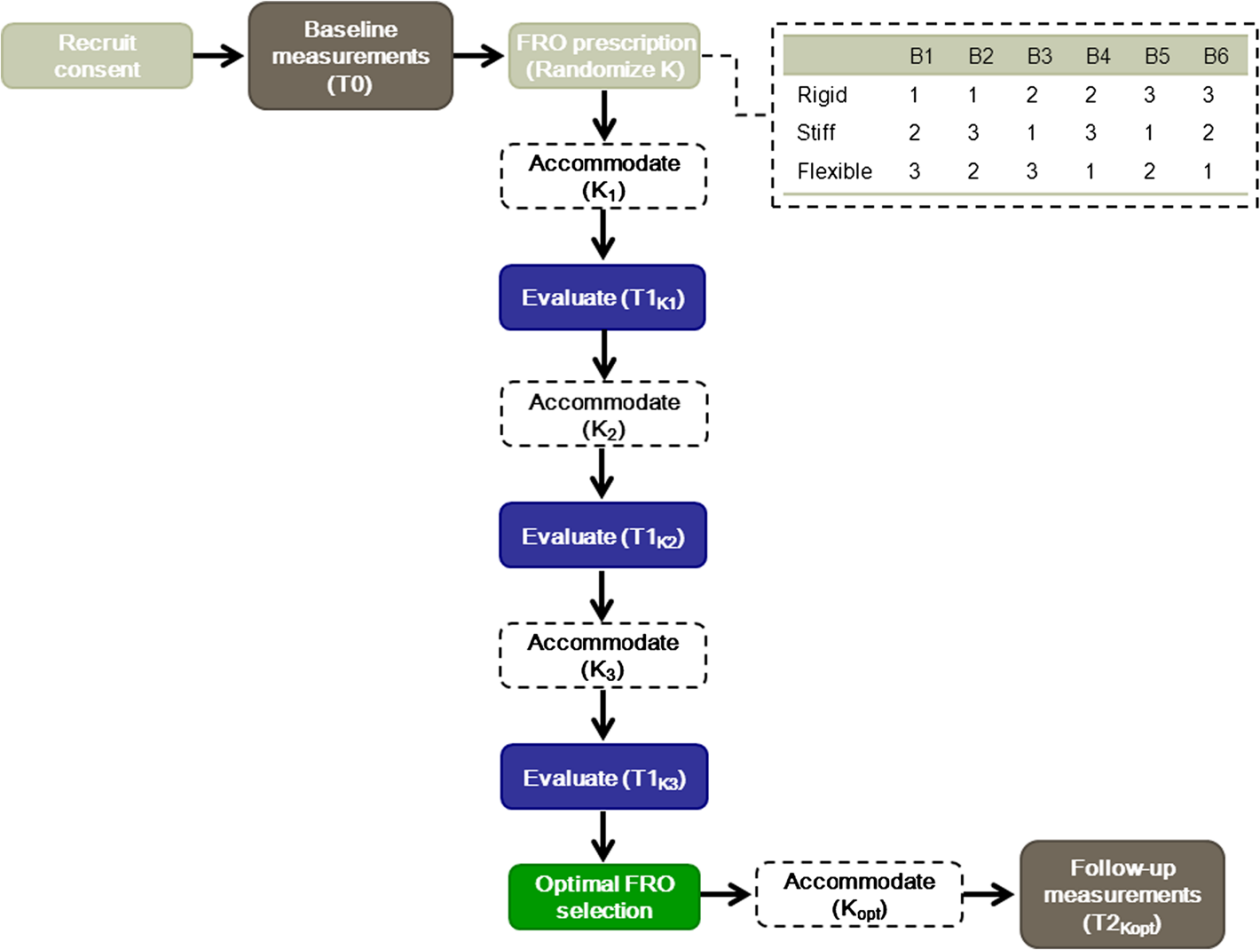
**Schematic representation of the study design. **Following baseline measurements (T0), the subject’s will be prescribed an interventional FRO. The stiffness of this FRO will be varied (rigid, stiff and flexible) and the order of FRO stiffness will be block randomised. Accommodation time for each stiffness will last 4–8 weeks, after which effects will be evaluated (T1_K1_, T1_K2_, and T1_K3_). Following these evaluations, an optimal FRO for the subject will be selected. Follow-up measurements (T2_Kopt_) will be carried out at 12–20 weeks. B=Block; FRO= Floor Reaction Orthosis; K=AFO stiffness; K_1_, K_2_, and K_3_ represent either rigid, stiff or flexible stiffness configurations.

A pre-post experimental study consisting of two repeated measurements, i.e. at baseline, T0, walking with shoes only (control), and at 12–20 weeks follow-up, T2_Kopt_, walking with an optimised FRO (case) will be performed to evaluate FRO efficacy in children with SCP (Figure 1). The study protocol has been approved by the Medical Ethics Committee of the VU University Medical Center in Amsterdam. 

Following completion of study enrolment, baseline measurements (T0) will be performed barefoot, with shoes only and with the subject’s current orthosis (if applicable). Stiffness (K) of the new FRO will be varied into three configurations: rigid, stiff, and flexible. A balanced block randomisation will be applied for six possible sequences of stiffness configurations, to ensure that the same number of patients is allocated to each sequence. Every configuration will be worn for an accommodation period of four to eight weeks, after which FRO efficacy will be evaluated (T1_k1_,T1_K2_ and T1_K3_). An analysis of the evaluation of all FRO configurations will allow the selection of the stiffness with the maximal benefit for a particular subject, referred to as the subject’s optimal FRO (the selection procedure is explained further below). Following this selection, the optimal FRO will be worn for twelve to twenty weeks, after which the follow-up measurements (T2_Kopt_) will be taken.

### Participants

#### Inclusion and exclusion criteria

Our aim is to include 32 children with SCP (Gross Motor Function Classification Score [42] (GMFCS) levels I, II and III [provided that the child is able to perform a 3D-gait analysis without walking aids]) who are candidates for a (new) FRO. Children will be recruited from the outpatient clinic of the VU University Medical Center, Amsterdam and affiliated rehabilitation centres.

Study information will be provided to potential participants in the form of a patient information letter and a brochure. Patients and parents willing to participate will be contacted by the primary investigator (YK), who will verify inclusion and exclusion criteria (Table 1). When a patient meets the inclusion criteria, oral and written informed consent will be obtained from both parents, and from children aged 12 years and older, in accordance with the declaration of Helsinki. 

**Table 1:** Inclusion and exclusion criteria

	
**Inclusion criteria**	
	Spastic CP;
	6-14 years;
	A gait pattern characterised by excessive knee flexion (jump gait, apparent equinus or crouch gait) [42];
	GMFCS I, II, or III (provided that the patient is able to walk independently for at least 15 meters)
**Exclusion criteria**	
	Any orthopaedic surgery or other surgical interventions that might influence mobility in the past 6 months;
	Botulinum toxin A injections in the past 3 months, Intrathecal Baclofen therapy in the past 6 months, or SDR in the past year;
	Impairments that could contraindicate fitness testing;
	Plantar flexion contractures or knee contractures >10° or hip endorotation > 20° in midstance;
	Other medical conditions influencing mobility;
	Severe behavioural problems;

GMFCS: Gross Motor Function Classification System [43]; SDR: Selective Dorsal Rhizotomy.

#### Sample size

The sample size will be based on a power analysis of the expected changes (i.e. T0 versus T2) in the primary outcome, walking energy cost [J^-1^⋅kg^-1^⋅m^1^. According to literature, walking energy cost in children with CP may be 30-50% higher than in healthy children [10-12]. SCP children with GMFCS levels I, II and III show a mean net EC of 5.02 (±1.70) J^-1^⋅kg^-1^⋅m^1^[26]. A reduction of 25% in this value (≈1.26 J^-1^⋅kg^-1^⋅m^1^) is considered to be a clinically significant change [25,26]. Assuming a power of 80% and a significance level of 0.05, detecting a clinically significant change will require a sample size of 29 children [44]. Allowing for a dropout of approximately 10%, a sample size of 32 will be sufficient.

### Investigational AFO

Investigational FROs will be composed of prepreg carbon, manufactured using the Mälmo-technique (Otto Bock HealthCare GmbH, Duderstadt, Germany). For fair evaluation of efficacy, the investigational FRO will be fabricated with a rigid footplate. To further ensure a fair comparison, tuning of the FRO-footwear combination following the Owen method will be carried out for each configuration [45].

Investigational FROs will be fabricated with an integrated Neuro Swing® system hinge (Fior & Gentz, Lüneberg, Germany), which is available in different sizes. The size of the hinge is dependent on the body weight and length of the patient. For this study, it is expected that only the 14mm and 16mm hinges will be used. The hinge holds an anterior and posterior shaft, and comes with a package of five springs, each with a different degree of stiffness. Ankle stiffness can be adjusted within the same orthosis, using different spring forces towards plantar and dorsal flexion. In this study, the hinge will be prepared in three configurations: rigid, stiff and flexible. The rigid configuration (i.e. ±4.3 Nm/deg) will entirely prevent dorsal or plantar flexion. For the stiff and flexible configurations, the spring force for dorsal flexion will be varied using the strongest spring (i.e. ±1.2 Nm/deg [14mm] and ±2.4 Nm/deg [16mm]) and the second strongest spring (i.e. ±0.5 Nm/deg [14mm] and ±1.0 Nm/deg [16mm]), respectively. The spring force towards plantar flexion will be very compliant (i.e. ±0.01 Nm/deg [14mm] and ±0.04 Nm/deg [16mm]) for both configurations.

#### Accommodation procedures

The accommodation period for all three FRO configurations will include a gradual increase in the length of time the FRO is worn each day, in order to minimise the risk of adverse events. Patients will be contacted one week after setting each new FRO configuration, to check for adverse events such as pain, discomfort, or pressure sores. If the patient has no complaints, the accommodation period will continue until the next visit (four to eight weeks later). When adverse events are reported, the investigator will identify the causes and make an appropriate decision according to protocol. The accommodation period will not start until all complaints are resolved.

#### Optimal AFO selection procedure

**Figure 2:**
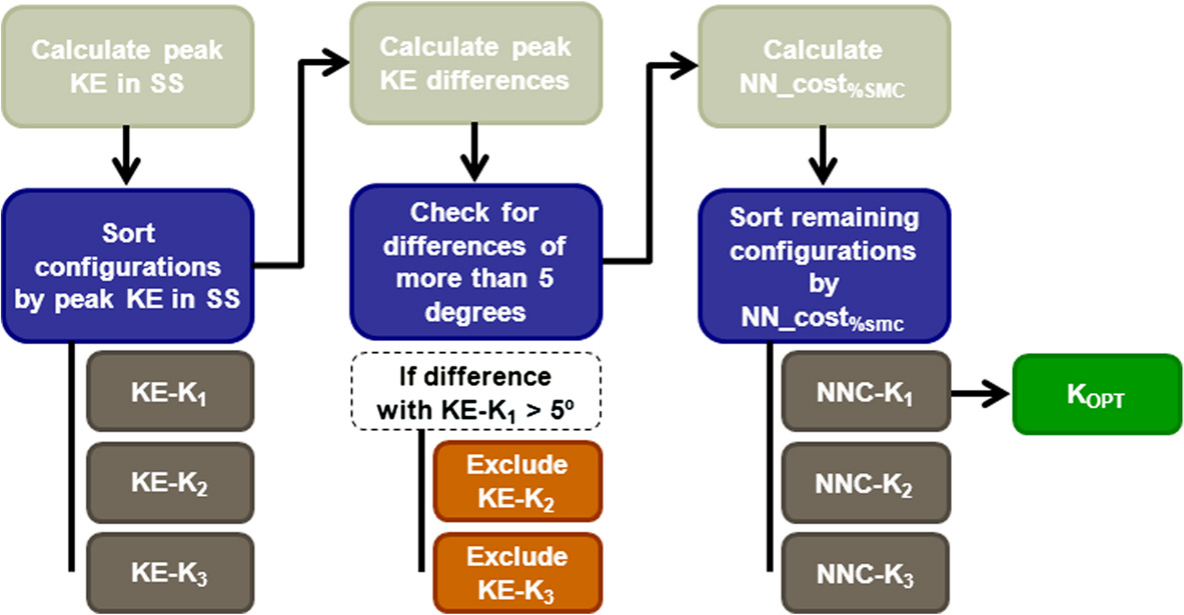
**Flowchart of the optimal FRO stiffness selection procedure. **After sorting the different stiffness configurations based on peak knee extension angle in single support (KE-K_x_), absolute differences in peak KE will be calculated. KE-K_2_ and/or KE-K_3_ will be excluded if this difference is more than five degrees. Otherwise, the remaining configurations will be sorted by net non-dimensional walking energy cost (NNC-K_x_) (this can be either two or three remaining configurations). The stiffness that results in the lowest walking energy cost will be selected as the subject’s optimal FRO. K_1,_ K_2_ and K_3_ = rigid, stiff, or flexible FRO stiffness configurations; K_OPT_ = subject’s optimal FRO stiffness; KE = knee extension angle; KE-K_x_ = stiffness sorted by KE; NN_cost_%SMC_ = net non-dimensional walking energy cost relative to speed matched controls; NNC-K_x_ = stiffness sorted by NN_cost_%SMC_; SS = single support.

Following a standard procedure, evaluation of FRO efficacy of the three configurations (T1_k1_, T1_K2_ and T1_K3_) will lead to selection of the subject’s optimal FRO configuration (Figure 2). Since clinical assessment of FRO effectiveness in children walking with excessive knee flexion is mainly based on knee kinematics in stance, the minimum amount of knee flexion (i.e. peak knee extension) in the single support phase will be the main discriminating parameter. The configuration that results in smallest peak knee flexion will be selected as the subject’s optimal FRO. Differences of less than 5º will be considered equal, since this angle lies within the variability of 3D gait analysis [46]. Should minimum knee flexion in single support be unable to discriminate between the remaining configurations, walking energy cost (expressed as net non-dimensional energy cost relative to speed-matched control cost (NN_EC_%SMC_) [47,48] will be decisive. In this situation, the FRO that results in the lowest NN_EC_%SMC_ will be selected as the subject’s optimal FRO. 

### Outcome measures

Outcome measures for this study are categorised in accordance with the ICF [34] and cover the components ‘body functions and structures’ and ‘activities and participation’, as well as personal and environmental factors. An overview of all outcome measures is presented in Table 2. 

**Table 2:** Overview of tests performed at different measurement moments

		**T0**	**T1**_k_*	**T2**_**kopt**_
**Primary study parameters**				
*Activities and participation*	ECWT	x	x	x
**Secondary study parameters**				
*Body functions and structures*	3D-gait analysis	x	x	x
*Activities and participation*	SAM***	x	x	x
	CAPE***	x		x
**Effect modifiers**				
*Body functions and structures*	Physical fitness test	x		
*Environmental and personal factors*	Physical examination**	x		
	Gait pattern	x		
	Intake questionnaire	x		
	BSS	x		
	FMS	x	x	x
	FAQ	x	x	x
	GMFCS	x		
***Other outcomes***				
	GAS	x		x
	FRO properties	x	x	x
	Motivation diary	x	x	x
	@ monitor		x	x
	Satisfaction	x	x	x

*T1_k_ will be repeated for each FRO-configuration: rigid, stiff and flexible.

** The physical examination includes passive Range of Motion, selective motor control and gross motor function tests.

***SAM and CAPE data will be assessed in the week prior to the ticked measurement moment.

BSS=’Bronnen van Steun en Spanning’; CAPE=Children’s Assessment of Participation and Enjoyment; ECWT=Energy Cost of Walking Test; FAQ=Functional Assessment Questionnaire; FMS=Functional Mobility Scale; FRO=Floor Reaction Orthosis; GAS=Goal Attainment Scaling; GMFCS=Gross Motor Function Classification System; SAM=StepWatch3^TM^ Activity Monitor.

#### Primary outcome

Our primary outcome measure is walking energy cost, which will be measured during a 6-minute walking test on an indoor oval track. Subjects will be asked to walk at a self-preferred comfortable speed, during which oxygen uptake and carbon dioxide production will be measured using the accurate and reliable Metamax 3B portable gas analysis system (Cortex Biophysik, Leipzig, Germany). Calculations will be based on measurements during a steady state of walking, defined as a period of at least one minute in which fluctuations in walking speed, oxygen uptake and carbon dioxide production show the least change [48].

Mean steady-state breath-by-breath oxygen uptake values and respiratory exchange ratios will be computed. Using these values, gross and net energy consumption will be calculated and normalised according to the net non-dimensional (NN) scheme of Schwartz et al. [48]. The primary outcome measures will be expressed as net energy cost and as NN energy cost as a percentage of speed-matched controls (NN_EC_%SMC_). Furthermore, non-dimensional walking speed (N_speed) (a secondary outcome measure) will be calculated.

EC measurements in children with CP are sufficiently sensitive, as shown by Brehm et al. [49]. The NN normalisation scheme of Schwartz et al. is suggested to be the preferred method for reporting oxygen consumption data for subjects who have not reached their full stature, since it is largely independent of mass, height and age [48].

#### Secondary outcome

Secondary outcome measures include daily activity, gait biomechanics, walking speed (N_speed) and diversity, intensity and enjoyment of participation (assessed with the Children’s Assessment of Participation and Enjoyment [CAPE]). Two of these outcome measures (daily activity and gait biomechanics) are further explained below.

##### Daily activity

Daily activity will be measured for one week with a StepWatch3™ Activity Monitor 3.0 (SAM) (Cyma Corporation Seattle, WA, USA), which is an ankle worn accelerometer that measures the average amount of steps per minute over a broad spectrum of cadences. The SAM will be attached to the ankle of the dominant leg. Subjects will be instructed not to remove the SAM at any time, except when taking a bath or shower or when swimming. For adequate interpretation of the data, subjects will be asked to keep a diary of their activity program during each day of the week.

Daily activity will be determined as 1) average total steps per day, 2) percentage of time children were active, 3) percentage of time children were inactive, 4) ratio of medium to low activity levels and 5) percentage of time children show high activity levels. A calibrated SAM has been shown to be an accurate tool for recording daily steps in children with CP [50,51].

##### Gait biomechanics

Joint kinematics will be assessed in the laboratory, using a three-dimensional (3D) motion analysis system (OptoTrak, Northern Digital, Waterloo, Canada), while the subject walks on a 10m walkway at a self-preferred comfortable speed. Marker clusters will be attached to the feet, shanks, thighs, pelvis and trunk. To determine anatomical coordinate systems, anatomical landmarks will be palpated according to Cappozzo et al. [52]. Joint kinetics will be calculated by assessment of the ground reaction force, using an integrated force plate (AMTI OR6-5-1000, Watertown, MA, USA).

At baseline, all subjects will be measured walking bare foot and with shoes only. An additional condition (old FRO-footwear combination) will be included for children who have (suitable) old orthoses. Follow-up recordings will be made while walking with the new FRO-footwear combination. Six trials, with the subject stepping on the force plate, will be completed for each condition (i.e. three trials for each leg). Data on joint kinematics, and kinetics around the hip, knee and ankle will be averaged. Spatio-temporal parameters, such as step length [m], step width [m] and cadence [steps⋅min^-1^] will also be calculated.

#### Effect modifiers

As potential effect modifiers, the following outcome measures will be assessed: demographic variables, disease characteristics, personal and family characteristics, level of functional mobility and physical fitness (explained below).

##### Physical fitness

Physical fitness will be measured by means of an aerobic and anaerobic exercise test on a bicycle ergometer. The aerobic test will be performed according to the protocol described by Balemans et al. [53] and aerobic fitness will be defined as oxygen uptake over the 30 seconds with the highest sustained load (VO2_peak_) [ml^-1^⋅kg^-1^⋅min^-^1]. Anaerobic power will be determined using the 20 seconds Wingate Anaerobic cycling Test (20s-WAnT), a sprint test against a constant breaking torque [54]. Anaerobic fitness will be defined by the mean anaerobic power over 20 seconds (P20_mean_) [W⋅kg^-1^ and by the highest power output within the 20 seconds, the peak anaerobic power (P20_peak_) [W⋅kg^-1^. Measurement procedures, equipment and protocols for both tests will be as described by Balemans et al. [53].

#### Other outcomes

Other study outcome measures will include 1) the patient’s personal treatment goals, measured with Goal Attainment Scaling (GAS), 2) treatment adherence, assessed with a motivation diary and with the @monitor [55], 3) satisfaction with the FRO, as perceived by the patient and parents and 4) FRO stiffness, measured with BRUCE, which is a recently developed device for measuring mechanical AFO properties [56].

### Statistical analysis

#### Subject population

Demographic variables and disease characteristics will be summarised using descriptive statistics. Furthermore, the means, medians, standard deviations and 95% confidence interval (CI) of primary and secondary outcome measures will be presented for all visits. In addition, correlations between parameters will be examined using correlation coefficients and graphical techniques.

#### Evaluation of FRO efficacy

Evaluation of the efficacy of a subject’s optimal FRO will be based on analyses of pre/post-intervention differences in primary and secondary outcome measures. The pre-intervention (control) condition will be for shoes only. Mean data for these measurements (assessed at T0) will be compared to follow-up measurements (T2_Kopt_), using paired sample t-tests.

To identify working mechanisms, multivariate linear regression analyses will be applied to investigate which of the changes in gait biomechanics are associated with changes in walking energy cost (model 1) and daily activity (model 2). First, a univariate regression analysis (ANOVA) will be performed to determine which factors are significantly associated with changes in the biomechanics of gait (p≤0.1), followed by the analysis of significant factors (p≤.05) in a multivariate regression analysis model.

#### Identifying prognostic factors

Multivariate regression analysis will also be applied to investigate to what extent child characteristics and FRO stiffness represent determinants for success of FRO treatment, defined as decreased walking energy cost (model 1), improvement in daily activity (model 2) and positive GAS scores (model 3). Initially, a univariate regression analysis (ANOVA) will be performed to determine which factors are significantly associated with FRO treatment outcomes (p≤0.1). Significant factors (p≤.05) will then be included in a multivariate regression model. Model analysis will include factors such as level of physical fitness, baseline disease characteristics, gait pattern, level of functional mobility, environmental factors and FRO characteristics.

## Discussion

This study will evaluate the effects of varying degrees of FRO ankle stiffness on different aspects of gait. Based on earlier studies, an optimal match is expected between specific patient characteristics and FRO stiffness. Assuming that there is an optimal FRO stiffness for each subject, this study might lead directly to an optimised FRO treatment for these patients. In addition, the study will evaluate FRO efficacy, using outcome measures that are relevant in the patient’s daily life (i.e. walking energy cost and daily activity), thereby emphasising clinical relevance.

Because the stiffness of an FRO should be based on the specific gait deviations of the child, the inclusion criteria of this study will be specifically defined. This will result in a relatively homogeneous study population, enabling a fair comparison of subjects. On the other hand, these strict criteria may make it difficult to generalise results to the wider treatment and prescription of FROs, also because the design of the investigational FRO design differs from conventional FROs. Nonetheless, it is expected that the results of the study will allow an optimal FRO treatment to be defined in this specific patient group.

This study will be the first to investigate broadly the efficacy of an individually optimised FRO, including evaluation of effects on multiple ICF levels. This will result in a unique data set with which to assess mutual relations between outcome measures. We anticipate that this analysis will aid in identifying both the underlying working mechanisms of FRO and the factors important to treatment success. In conclusion, the data generated by this study may provide not only novel insights, but may also contribute to improved FRO treatment in SCP in the (near) term.

## Abbreviations

AFO: Ankle-Foot Orthosis; FRO: Floor Reaction Orthosis; GMFCS: Gross Motor Function Classification System; ICF: International Classification of Functioning, Disability and Health; ROM: Range of Motion; SCP: Spastic Cerebral Palsy.

## Competing interest

The authors declare that they have no competing interests.

## Authors’ contributions

All listed authors contributed to the conception and design of the study. All authors were involved in drafting the manuscript and read and approved the final manuscript. All co-authors and contributors have approved the acknowledgement of their contributions.

## Pre-publication history

The pre-publication history for this paper can be accessed here:

http://www.biomedcentral.com/1471-2431/13/17/prepub
